# Simultaneous occurrence of transient global amnesia and Takotsubo syndrome triggered by caring for a terminally ill relative

**DOI:** 10.1002/ccr3.8797

**Published:** 2024-04-24

**Authors:** Claudia Stöllberger, Josef Finsterer

**Affiliations:** ^1^ Office Wehlistraße Vienna Austria; ^2^ Neurology and Neurophysiology Center Vienna Austria

**Keywords:** emotional stressor, hypothyroidism, migraine, Takotsubo syndrome, transient global amnesia

## Abstract

Takotsubo syndrome and transient global amnesia can occur simultaneously, not only in the context of acute but also long‐standing emotional stress. Probably, hypothyroidism and migraine make the patient more susceptible to both of these disorders.

## 1 INTRODUCTION

Transient global amnesia (TGA) is characterized by a sudden, complete inability to retain new information, lasting for several hours, with preservation of alertness and all other cognitive functions. During TGA, new information can only be retained for more than a few seconds, and retrograde amnesia extends backward for several hours, days, or longer.^1^ Takotsubo syndrome (TTS) is characterized by acute transient left ventricular systolic dysfunction, not explained by coronary artery disease, which has to be distinguished from acute myocardial infarction.^2^ Emotional and physical stressors play a role as triggers for TGA and TTS, and coincidence of both conditions has been reported (Table 1).^3^, ^4^, ^5^, ^6^, ^7^, ^8^, ^9^, ^10^, ^11^, ^12^, ^13^, ^14^ In a cross‐sectional study of US hospitalizations from 2006 to 2014, there were 155,105 diagnoses of TTS. TTS was associated with TGA with an odds ratio of 2.3 (95% confidence interval 1.5–3.6).^15^ In most cases, the trigger lasted only for a short period of time (Table 1). We report a case of simultaneous TGA and TTS triggered by a long‐term stressful condition.

**TABLE 1:** Type and duration of triggers in patients with TTS and TGA (included were only reports which described the triggering event).

Author	Year of Publication	Age/sex	Trigger	Start of the trigger before onset of TTS
Stöllberger^3^	2010	77/f	Assault	Few hours
Grautoff^4^	2012	69/f	Health problems of the daughter	24 h
Bobinger^5^	2013	62/f	Visit to a public swimming pool	Few hours
Toussi^6^	2014	66/f	Witnessing sister's death	Immediately
Quick^7^	2015	57/f	Son died in a car accident	2 weeks
Sajeev^8^	2017	60/f	Anniversary of the death of a loved one	Few hours
Stöllberger^9^	2018	57/f	Existential fear	1 week
Pyle^10^	2018	59/f	Anxiety attack	Few hours
Finsterer^11^	2019	64/m	Funeral of brother	Few hours
Tso^12^	2019	62/f	Death of a family member, recent family court case	1 year
Papadis^13^	2022	75/f	Job meeting	Immediately
Dziewierz^14^	2023	64/f	Acute emotional stress event	Few hours

Abbreviations: TGA, Transient global amnesia; TTS, Takotsubo syndrome.

## 2 CASE HISTORY/EXAMINATION

A 67‐year‐old female in general good health with a body mass index 26.4 kg/m^2^, with a history of hyperlipidemia, migraine without aura, mild depression, and hypothyroidism started to suffer from headache during training at the gym with light weights (10 lb and 12 lb) for arms, chest, and legs. She felt nauseous and called her husband. When he came to the gym, he asked her if she was well enough to drive. She said yes, but couldn't remember where she parked the car. Her husband was concerned because she kept repeating herself, saying that she did not feel good. She did not remember anything that happened for the next 8 h. She did not remember that her sister had died 3 months ago and did not know what day, month, or even year it was. The husband took her to the hospital because he suspected myocardial infarction or stroke.

During the 18 months before this event, she was taking care of her sister, who suffered from ovarian cancer. She was with her 3–4 times a week until the last 8 weeks, when she was there each day. She was also with her when she died. Afterwards, she got the sister's estate settled, sold the home, and helped her daughters through all of this.

She was on a chronic medication with bupropion 200 mg/d, levothyroxine 75 μg/d, rosuvastatin 5 mg/d, butalbital 50 mg/d, acetaminophen 325 mg/d, and caffeine 40 mg/d.

## 3 METHODS (DIFFERENTIAL DIAGNOSIS, INVESTIGATIONS AND TREATMENT)

In the emergency department, her heart rate was 89/min, respiratory rate was 20/min, blood pressure was 206/107 mmHg, and O_2_ saturation was 98%. Laboratory tests showed an initially elevated high‐sensitivity troponin of 138 ng/L, which increased to 1344 ng/L (normal range: 0–54 ng/L). The thyroid stimulating hormone (TSH) level was 1.6 μU/mL (normal range: 0.27–4. μU/mL). The electrocardiogram showed sinus rhythm with repolarization abnormalities.

Computed tomography (CT) of the head showed no acute intracranial abnormality; CT angiography of head and neck showed no stenosis, signs of vasoconstriction, aneurysm, dilatation, or dissection within the intracranial or extracranial arterial circulation. Magnetic resonance imaging (MRI) of the brain showed no acute infarct and no hippocampal foci of diffusion restriction.

She underwent cardiac catheterization with no evidence of coronary artery disease. The left ventricular ejection fraction (LVEF) was 50%, and anterolateral wall hypokinesia was seen. Cardiac MRI showed a normal left ventricular cavity size and global systolic function with a LVEF of 57% and a mild hypokinesia involving the basal to mid‐anterior wall. Additionally, faint edema and mild mid‐myocardial/epicardial delayed enhancement involving the basal to mid‐anterior and septal segments were seen, but there was no evidence of myocardial infarction. Right ventricular cavity size and systolic function were normal.

Clinical investigation 20 h after onset of symptoms found her seated on the bed, awake and alert. She had no complaints, denied symptoms of headache and vision changes. The memory was back to normal. She denied symptoms of tingling, numbness, palpitations, weakness, dizziness, or lightheadedness and walked with steady gait. Metoprolol 25 mg/d was added to her medication. She was discharged after 3 days.

## 4 CONCLUSION AND RESULTS (OUTCOME AND FOLLOW‐UP)

Before, during, and after hospital admission, she never felt anginal chest pain. Because of low blood pressure, her cardiologist reduced metoprolol to 12.5 mg/d. After discharge from the hospital, she started grief counseling and is doing much better mentally and physically. Four weeks after discharge, neither wall motion abnormalities nor repolarization abnormalities were visible in the echocardiogram and electrocardiogram. Based on the clinical and instrumental findings in the acute phase and the reversibility of systolic dysfunction in follow‐up, the diagnosis of TTS associated with TGA was established.^1^, ^2^

## 5 DISCUSSION

The pathogenic mechanisms behind TTS and TGA remain unsolved, but a catecholamine surge may be part of the underlying pathophysiology.^1^, ^15^ Based on several pathophysiologic findings, it has been suggested that TGA might be a “cerebral Takotsubo.”^16^

The presented patient suffered from two comorbidities that are known to be associated with TTS as well as TGA, migraine and hypothyroidism. Migraine has been identified as a risk factor for TTS as well as TGA.^1^, ^15^, ^17^ Hypothyroidism is more frequent in patients with TTS as well as TGA than in the general population.^17^, ^18^, ^19^ The pathomechanism of hypothyroidism as well as migraine in these disorders, however, is unclear. Although not exhausting, our patient underwent physical exercise during onset of symptoms. Exercise is an acknowledged trigger for TGA as well as TTS.^2^, ^20^

While depression has been identified as a risk factor for TGA, its role as a risk factor for TTS is controversial.^21^, ^22^ The coincidence of TGA and TTS with depression has, to our knowledge, not been described.

Acute dysregulation of blood pressure is a frequent finding in TGA patients without a history of arterial hypertension, like our patient.^23^, ^24^ It is hypothesized that these patients are obviously not adapted to high blood pressure episodes, which may act as a possible trigger for metabolic stress in the vulnerable hippocampal region causing TGA.^24^ The transient increase in serum troponin levels could be due to the acute rise in blood pressure as well as due to TTS.^2^, ^25^

The coincidence of TGA and TTS associated with a long‐lasting emotional stressor has, to our knowledge, only been described in one case.^12^ (Table 1) Of note, similar to our patient, the reported female suffered from hypothyroidism.

The comedication of our patient may possibly have contributed to the development of TTS. TTS has been reported after ingestion of a weight management supplement containing caffeine and amphetamine‐like stimulants.^26^ In a further case, TTS occurred after a combined drug intoxication containing acetaminophen.^27^

We conclude that TTS and TGA can occur simultaneously, not only in the context of acute but also chronic emotional stress. Probably, hypothyroidism and migraine make the patients more susceptible to TTS and TGA. The clinician should consider TGA in a patient with TTS, and TTS in a patient with TGA, and perform the respective diagnostic tests if there are symptoms of TTS in a patient with TGA and vice‐versa.

## AUTHOR CONTRIBUTIONS

**Claudia Stöllberger:** Conceptualization; data curation; investigation; methodology. **Josef Finsterer:** Formal analysis; investigation; methodology; writing – original draft; writing – review and editing.

## FUNDING INFORMATION

No funding and no grants.

## CONFLICT OF INTEREST STATEMENT

No conflict of interest (both authors).

## CONSENT

Written informed consent was obtained from the patient to publish this report in accordance with the journal's patient consent policy.

## Data Availability

Data pertaining to this case can be obtained by contacting the corresponding author.
